# Epigallocatechin-3-Gallate (EGCG) as a Multifaceted Natural Anticancer Agent in Colorectal Cancer: Preclinical Evidence and Future Directions

**DOI:** 10.3390/cancers18152391

**Published:** 2026-07-24

**Authors:** Nicolaos Achilleos, Maddox Talbot Harrison, Evangelia Tsiani

**Affiliations:** 1 Department of Health Sciences, Faculty of Applied Health Sciences, Brock University, St. Catharines, ON L2S 3A1, Canadamh21kq@brocku.ca (M.T.H.); 2 Centre for Bone and Muscle Health, Applied Health Sciences, Brock University, St. Catharines, ON L2S 3A1, Canada

**Keywords:** colorectal cancer, CRC, EGCG, apoptosis, proliferation, cancer therapy

## Abstract

Colorectal cancer (CRC) accounted for nearly 2 million new cases and over 900,000 deaths worldwide in 2020. Current treatment strategies include surgery, chemotherapy, radiotherapy, and targeted therapies, including monoclonal antibodies and other forms of immunotherapy. Despite these options, high mortality rates and frequent treatment-related adverse effects underscore the urgent need for novel therapeutic approaches. Epigallocatechin-3-gallate (EGCG), the most abundant catechin in green tea, exhibits potent antioxidant, anti-inflammatory, and anticancer activities. This review summarizes evidence from the past decade on the in vitro and in vivo effects of EGCG in CRC. In vitro studies demonstrate that EGCG inhibits cell proliferation and migration while inducing apoptosis through multiple molecular mechanisms, including modulation of signaling pathways such as Wnt/β-catenin, PI3K/Akt, and NF-κB. In vivo studies in mouse xenograft models indicate that EGCG reduces tumor growth, volume, and mass. Nevertheless, additional in vivo investigations are needed to fully define EGCG’s therapeutic potential and its translational relevance for human clinical trials.

## Simple Summary

Colorectal cancer (CRC) was the second leading cause of cancer-related mortality in 2020, and it is projected that its burden will increase by 2040. Current treatments face significant problems such as increased drug resistance and toxicity. Epigallocathechin-3-Gallate (EGCG), a catechin found primarily in green tea, has shown promising results against CRC both in vitro and in vivo. The main aim of this narrative review is to summarize current evidence for EGCG use in CRC and highlight the benefits of EGCG nanoformulations and EGCG combinations. Ultimately, the review of the current evidence will facilitate further in vivo and clinical research on the effects of EGCG, EGCG nanoformulations or EGCG combinations with other molecules in animals with CRC xenografts and CRC patients.

## 1. Introduction

### 1.1. Colorectal Cancer

Colorectal cancer (CRC) emerges from the epithelial lining of the colon or rectum. It is the third most commonly diagnosed cancer worldwide and the second leading cause of cancer-related mortality in 2020 [1]. In the same year, it accounted for approximately 1.9 million new cases and 935,000 deaths globally [1]. The global CRC burden is predicted to increase substantially by 2040 due to population aging and shifting exposure to modifiable risk factors [2].

CRC develops through accumulated genetic and epigenetic alterations that disrupt intestinal epithelial homeostasis and key signaling pathways [3,4]. Normally, crypt-base stem cell renewal is regulated by Wnt/β-catenin signaling, but adenomatous polyposis coli (APC) loss stabilizes β-catenin and drives transcription of proliferative genes such as MYC and CCND1 [3]. EGFR/RAS/MAPK and PI3K/Akt activation further promotes proliferation, metabolic adaptation, and apoptosis resistance, while loss of tumor suppressor protein p53 (TP53) weakens DNA damage responses and cell cycle control [3,4,5]. KRAS or BRAF mutations also contribute to uncontrolled growth and resistance to anti-EGFR monoclonal antibodies [5,6]. CRC heterogeneity has recently been refined through pathway-derived subtypes (PDS), which encompass PDS1, PDS2, and PDS3 tumors characterized by coordinated pathway activation patterns [7].

The gut microbiota has been increasingly recognized as a potential regulator of CRC development. The intestinal microbial ecosystem influences epithelial barrier integrity, immune activation, and metabolic signaling [3,4]. Dysbiosis of microbiota has the potential to promote tumorigenesis, primarily through chronic inflammation, oncogenic pathway activation, and production of pro-carcinogenic metabolites [3,4,8].

Inflammation is also a central contributor to colorectal carcinogenesis. Chronic inflammatory signaling can disrupt epithelial barrier integrity, sustain immune and stromal cell activation, and promote tumor initiation, progression, angiogenesis, invasion, and therapeutic resistance [9]. Inflammatory cytokine networks, including IL-6, IL-1β, TNF-α, and other mediators, regulate intestinal tissue homeostasis but can also support adenoma formation and tumor progression when persistently activated [10].

Both modifiable and non-modifiable factors can influence the development and prognosis of CRC. Modifiable risk factors include obesity, insulin resistance, type 2 diabetes mellitus, smoking, alcohol consumption, and diets rich in saturated fats. Non-modifiable risk factors include inflammatory bowel disease, male sex, and age greater than 65 years [4].

Localized CRC is commonly treated with surgical resection, while systemic therapies include fluoropyrimidines (5-fluorouracil and capecitabine), oxaliplatin, irinotecan, regorafenib, and trifluridine/tipiracil [5]. Radiotherapy is also used, often in combination with chemotherapy [11]. Biomarker-selected patients may benefit from targeted therapies, including anti-EGFR monoclonal antibodies, anti-VEGF agents, and immune checkpoint inhibitors [5,6]. However, surgery carries risks such as infection and venous thromboembolism; chemotherapy can cause neuropathy, myelosuppression, nausea, and ototoxicity; and radiotherapy may lead to enteritis and fibrosis [5,12]. Drug resistance further limits treatment efficacy, highlighting the need for complementary and preventive strategies [6,12].

### 1.2. EGCG

Plant-derived chemicals have been successful in cancer treatment, with drugs like the taxanes docetaxel and paclitaxel offering an effective solution for the treatment of different types of cancer [13,14]. Despite that, taxanes are not considered standard treatment options for CRC, and other plant-derivatives, such as irinotecan, which is an analogue of the natural substance and topoisomerase inhibitor camptothecin, are used as first-line options for CRC treatment [15]. In addition to direct anticancer effects, phytochemicals are increasingly investigated for their ability to modulate inflammation, enhance chemotherapy efficacy, and reduce treatment-related toxicity [16]. The use of these molecules in clinical practice highlights the importance and continued relevance of plant-derived molecules in oncology, thus providing a rationale for investigating their anticancer and anti-inflammatory properties.

**Figure 1:**
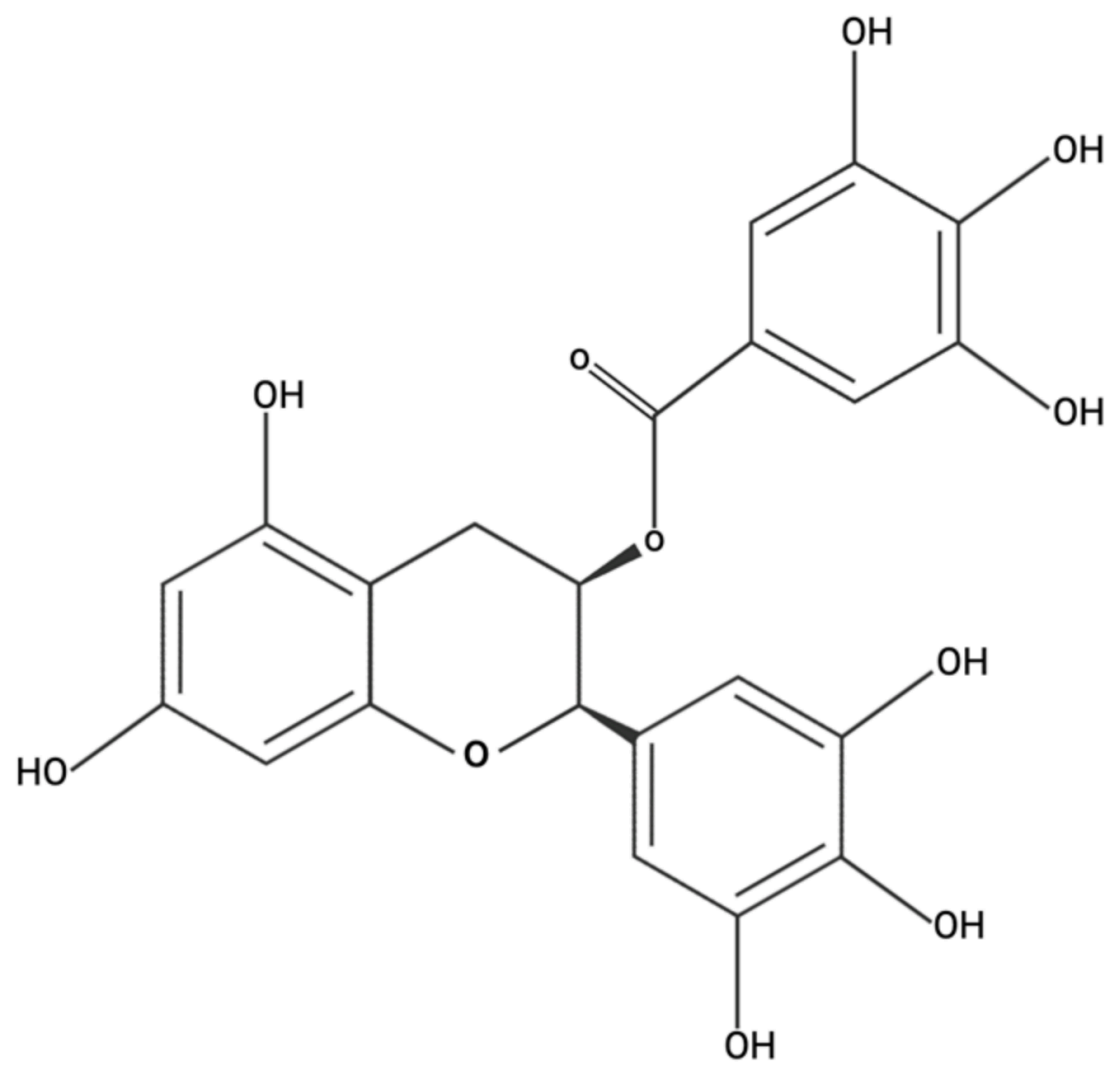
Chemical structure of epigallocatechin-3-gallate (EGCG). Created in Biorender. (Achilleos, N. (2026). https://BioRender.com (accessed on 15 November 2025)).

Green tea is composed of several catechins, primarily epigallocatechin-3-gallate (EGCG) (Figure 1), epigallocatechin (EGC), epicatechin gallate (ECG), and epicatechin (EC). EGCG comprises approximately 40–60% of total catechin content [17,18,19].

Catechins are flavonoids belonging to the flavan-3-ol subclass and contain a characteristic benzopyran core structure with multiple hydroxyl substitutions [20,21]. EGCG specifically contains a trihydroxylated B-ring and a gallate ester moiety. EGCG undergoes phase II metabolism, including methylation, glucuronidation, and sulfation, once ingested [22]. The gut microbiota contributes to catechin biotransformation, generating smaller phenolic metabolites that may retain biological activity [23,24].

**Figure 2:**
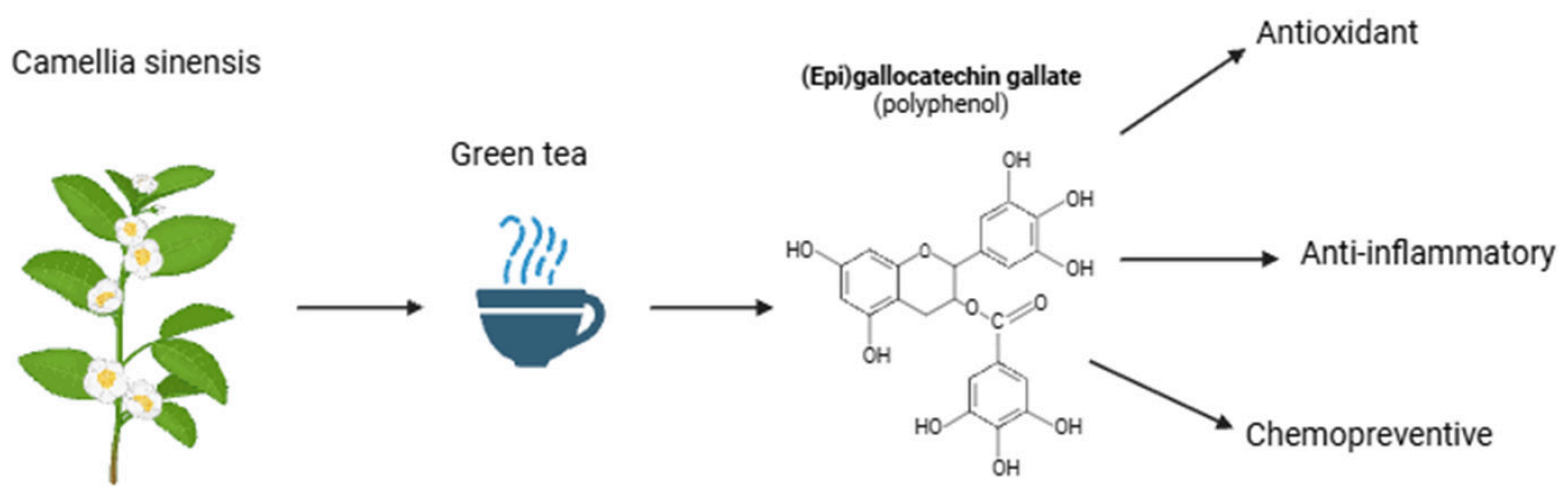
Green tea isolation and therapeutic properties of its most abundant catechin, EGCG. Created in Biorender. (Achilleos, N. (2026). https://BioRender.com (accessed on 15 November 2025)).

EGCG has been found to have antioxidant, anti-inflammatory, and chemopreventive effects (Figure 2) in lung, pancreatic, ovarian, and melanoma models through modulation of DNA methyltransferases, inhibition of Akt and STAT3 signaling, suppression of cancer stem cell phenotypes, and metastasis inhibition [18,25,26,27,28,29]. These broad-spectrum effects suggest that EGCG exerts pleiotropic antitumor and anti-inflammatory activity across multiple malignancies. These effects suggest that EGCG may help overcome some of the major limitations of conventional therapies and could serve as a complementary treatment strategy for colorectal cancer (CRC) and other malignancies.

This review is a narrative review that analyzes in vitro and in vivo studies and clinical trials conducted from 2009 to 2026 and examines the anticancer effects of EGCG in CRC. PubMed and Google Scholar were used to search for published articles in peer-reviewed journals. The date of the final search was 28 May 2026. The search terms used were “CRC” OR “colorectal cancer” AND “EGCG” OR “green tea”. The inclusion criteria set were the use of at least one well-established CRC cell line or CRC xenografted animal model and in vitro or in vivo use of pure EGCG treatment or green tea extract (GTE) with known EGCG concentrations if pure EGCG data were limited. Studies that did not fulfill these criteria were excluded from this review. All the authors of this review independently reviewed the titles and abstracts of all identified studies that were evaluated. After the titles and abstracts were deemed relevant to the topic, the authors evaluated the full texts to ensure fulfillment of the inclusion and exclusion criteria. The latest version of each study was used for evaluating the most updated and complete data. Because colorectal cancer is a multifaceted disease, the investigators of this review aimed to include studies that cover how EGCG affects different aspects of the disease, such as inflammation, cancer cell metabolism, proliferation, apoptosis, and migration. To our knowledge, there are two other reviews published in the last 5 years that have explored the effects of EGCG in CRC [30,31]. Randisi et al. [30] broadly evaluated green tea constituents, with a substantial emphasis on epidemiological studies, meta-analyses, and human green tea consumption, whereas Bao et al. [31] primarily organized EGCG evidence according to its biological effects and molecular mechanisms. In contrast, the present review provides a study-level synthesis of EGCG as a therapeutic candidate, separately evaluating free EGCG, EGCG nanoformulations, and EGCG combined with chemotherapy, radiotherapy, or other agents across preclinical and clinical evidence. By systematically reporting the specific cell lines and animal models used, treatment doses and durations, observed effects, and associated mechanisms, this review provides researchers and students with a comparative reference for evaluating differences across studies and identifying gaps that require further investigation.

## 2. Effects of EGCG Against Colorectal Cancer

### 2.1. EGCG Against Colorectal Cancer: In Vitro Studies

In HCT-116 CRC cells, EGCG (5 μM) significantly reduced viability, proliferation, and invasion (Table 1). These effects were associated with suppression of p-Met, p-ERK, and p-Akt by EGCG [32].

Jin et al. [33] used LoVo, HT-29, HCT-8, and SW480 CRC cells and showed that EGCG significantly inhibited proliferation and induced apoptosis and cell cycle arrest. Flow cytometry showed G0/G1 arrest in SW480 and LoVo cells, S-phase arrest in HT-29 cells, and G2/M arrest in HCT-8 cells. EGCG also downregulated Notch2 and HES1 in all cell lines, while Notch1 increased only in LoVo and SW480 cells. Notch2 and HES1 downregulation was associated with decreased proliferation of CRC cells. More studies are needed to determine the role and differences in expression of Notch1 in different CRC cell lines. These data suggest that EGCG inhibits the proliferation of CRC cells through Notch signaling regulation [33].

EGCG (12.5–100 μM) dose-dependently reduced proliferation and viability, while inducing apoptosis in RKO and HCT-116 CRC cells. These effects were associated with decreased Akt phosphorylation and VEGFR2 mRNA expression and increased p38 phosphorylation [34].

Chen et al. [35] cultured DLD-1 and SW480 CRC spheroids to better represent the tumour microenvironment and found that treatment with EGCG (10–60 μM) for 6 days reduced spheroid number and size, assessed by spheroid formation assay. EGCG downregulated colorectal cancer stem cell (CSC) markers CD133, CD44, ALDHA1, Oct-4, and Nanog at protein and mRNA levels. Reduction in CSC markers and spheroid formation is associated with decreased metastasis, drug resistance and malignancy recurrence. EGCG also reduced p-GSK3β, β-catenin, c-Myc, cyclin D1, and PCNA, while upregulating GSK3β. With EGCG treatment, Bcl-2 expression was downregulated, and Bax, caspase-3, caspase-8, and caspase-9 expression was upregulated, indicating EGCG-mediated apoptosis induction. GSK3β upregulation was essential for EGCG’s effects since GSK3β inhibition reduced EGCG-mediated inhibition of Wnt/β-catenin signaling, spheroid formation, and CD133 expression [35].

In HT-29 CRC cells, EGCG reduced viability and induced apoptosis, with no toxicity in normal 3T3 embryonic fibroblasts, suggesting sparing of normal/healthy tissue. EGCG increased caspase-3/7 activation, indicating apoptosis induction. EGCG also increased BiP, PERK, p-eIF2α, ATF4, and IRE1α, all markers of endoplasmic reticulum (ER) stress [36].

Treatment with EGCG increased transferrin receptor (TfR) and decreased ferritin heavy chain (FtH) in HT-29 CRC cells, indicating iron depletion and reduced CRC-related iron overload. Computational molecular docking showed strong EGCG–FtH binding affinity, suggesting that direct FtH inhibition may contribute to FtH downregulation and reduced CRC cell survival. These data highlight the iron-chelating properties of EGCG and associate them with decreased CRC cell survival. Despite being promising, however, further studies are required to examine whether EGCG could indeed bind to FtH [37].

Zhang et al. [38] found that EGCG decreased proliferation and viability, induced G1 arrest, increased p21 and p53, and decreased cyclin D1. EGCG also induced apoptosis, shown by flow cytometry and TUNEL staining, and increased caspase-3 and caspase-9 cleavage, autophagic vacuoles, beclin-1, and LC3B. Microarray and RT-qPCR showed altered expression of AKR1C1, IFI6, XAF1, PTEN, VEGFA, IL-8, and TMEM97. Gene Ontology (GO), Kyoto Encyclopedia of Genes and Genomes (KEGG), and liquid chromatography–mass spectrometry (LC-MS) analyses showed effects on cholesterol biosynthesis, NADP binding, steroid biosynthesis, glutathione metabolism, fatty acid metabolism, carbohydrate metabolism, terpenoid backbone biosynthesis, and glycerophospholipid metabolism. Overall, EGCG altered the transcriptomic and metabolomic profiles in HT-29 CRC cells [38].

EGCG reduced proliferation of the human CRC cell lines SW480, SW620, and LS411N. EGCG increased apoptosis, reduced migration in scratch-wound and transwell assays, and disrupted mitochondrial membrane potential. These effects were associated with increased cleaved caspase-3 and PARP; reduced Bcl-2, Bim, MCL-1, STAT3, and p-STAT3; and EMT-related changes, including increased E-cadherin and decreased vimentin. EGCG also reduced STAT3 promoter activity and mRNA levels in SW480 cells. STAT3 induces the proliferation and migration of CRC cells; thus, this study suggests that EGCG reduces proliferation and migration through STAT3 inhibition [39].

EGCG, in HCT116 and HT-29 CRC cells, reduced viability in a dose- and time-dependent manner, assessed by MTT assay, with no effect in normal 3T3-L1 fibroblasts. EGCG reduced lipid droplet accumulation, lipid droplet size, and intracellular/extracellular triglycerides. Treatment with EGCG reduced expression of key lipid metabolism markers involved in fatty acid synthesis (FASN, ACC, SCD1, ACLY, and SREBP1c), lipid uptake (CD36/FAT and FATP4), fatty acid β-oxidation (CPT1A, ACOX1, and PPARα) and mitochondrial biogenesis (UCP1 and PGC-1α) while increasing ATGL and HSL, two key lipolysis enzymes. EGCG reduced OCR and ECAR and increased AMPK Thr172 phosphorylation, suggesting AMPK-mediated regulation of lipid metabolism and viability [40].

Chen et al. [41] observed that EGCG reduced proliferation, migration, and cancer-associated fibroblast (CAF) activity in HCT116 and HT-29 CRC cells. CAFs were generated by co-culturing human intestinal fibroblasts (HIFs) with HCT116 and HT-29 cells, with CAF transformation shown by altered morphology and increased α-SMA, ACTA2, and FAP, key tumor microenvironment markers. EGCG dose-dependently inhibited CRC cell proliferation and migration. Furthermore, EGCG reduced the expression of aerobic glycolysis-associated markers, including lactate, PFK, and MCT4, and attenuated their ability to enhance CRC cell proliferation, migration, and invasion [41].

In HCT116 and HT-29 CRC cells, EGCG inhibited proliferation, induced apoptosis, disrupted mitochondrial membrane potential, and reduced MMP, ATP, ACC levels and fatty acid production. Palmitate (100 μM) attenuated EGCG-induced apoptosis in HCT116 but not HT-29 cells, suggesting fatty acid synthesis-dependent apoptosis in HCT116 cells. These data suggest that EGCG triggers apoptosis by suppressing de novo lipogenesis in CRC cells [42].

SW480 CRC cells treated with EGCG showed reduced cell viability and invasion. EGCG decreased STAT3 and CXCL8 in SW480 cells co-cultured with EGCG-treated neutrophils, leading to apoptosis induction and inhibition of CRC cell migration and invasion. Phorbol ester (PMA)-induced neutrophil extracellular traps (NETs) or STAT3 overexpression reduced these effects. EGCG also decreased the NET markers MPO and H3Cit, which was associated with EGCG-mediated STAT3 inhibition and further suppression of CRC cell migration and invasion. These findings suggest that EGCG may suppress neutrophil-mediated inflammatory signaling in the CRC tumor microenvironment through STAT3/CXCL8 and NET inhibition [43].

Wang et al. [44] found that EGCG (25–125 μM) increased LATS1/2 and p-LATS1/2 n RKO, HCT-116, SW620, and LoVo CRC cells. In HCT-116 and RKO cells, EGCG increased YAP, decreased YAP Ser127/Ser381 phosphorylation, promoted YAP nuclear localization, and increased CTGF, CYR61, and ANKRD1. YAP knockdown enhanced EGCG-mediated growth inhibition, apoptosis, ROS accumulation, migration reduction, and drug efflux suppression. Decreased migration was associated with increased E-cadherin and ZO-3 expression and reduced levels of N-cadherin, vimentin, Snail, and Slug, while inhibition of drug efflux was evidenced by the downregulation of the transporters ABCB1 and ABCC1 [44].

In CaCo2 CRC cells, >10 kDa EGCG polymers generated by autoxidation and ultrafiltration centrifugation reduced viability dose-dependently. Treatment with EGCG polymers induced apoptosis, which was associated with ACE, AT1R, and AngII downregulation and ACE2 and AGT upregulation. EGCG polymers also increased the pro-apoptotic markers γ-H2AX, Bax/Bcl-2 ratio, cleaved PARP, and p-p53. Overall, these data suggest that EGCG oxidation-derived polymers induce apoptosis in CRC cells through renin–angiotensin system regulation [45].

EGCG induced apoptosis, tumor microenvironment alterations, and immunogenic cell death (ICD) in SW480, HT-29, CT26, and MC38 colon cancer cells [43]. EGCG reduced viability dose-dependently and increased ICD-associated damage-associated molecular patterns (DAMPs), including HMGB1, CRT, and HSP70, assessed by ELISA, flow cytometry, and 7-AAD staining. EGCG was also found to alter the tumor microenvironment by promoting CD80 activation, which was associated with increased dendritic cell (DC) infiltration and enhanced production of IFN-γ and TNF-α by tumor-infiltrating CD8^+^ T cells. These findings support an immunomodulatory role for EGCG, suggesting that it may reshape the CRC inflammatory tumor microenvironment toward antitumor immune activation. In HT-29 and SW480 cells, EGCG increased the mRNA levels of the ER stress markers ATF3, ATF4, and DDIT3, suggesting ER stress-mediated ICD induction [46].

Suetsugu et al. [47] cultured CaCo2, CW-2, COLO-320, LoVo, and WiDr CRC cells and showed that EGCG reduced proliferation only in CaCo2 and CW-2 cells [44]. In CW-2 cells, EGCG increased apoptosis, shown by elevated caspase-cleaved cytokeratin 18 levels. EGCG also increased receptor tyrosine kinase phosphorylation, especially TrkB, and downregulated miR-187-5p. Further studies are needed to clarify the roles of TrkB and miR-187-5p in CRC [47].

**Figure 3:**
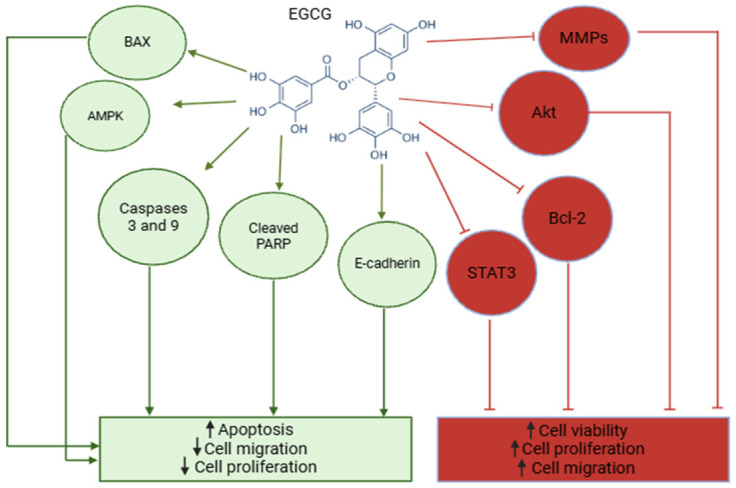
Summary of the effects of EGCG in human colorectal cancer cells. EGCG induced apoptosis and decreased cell migration and cell proliferation, which effects were associated with inhibition of STAT3, Bcl-2, Akt, and MMPs and increased E-cadherin, cleaved PARP, caspases, AMPK, and Bax. Green arrows show stimulation; red arrows show inhibition. ↑ increased, ↓ reduced. Created in Biorender. (Achilleos, N. (2026). https://BioRender.com (accessed on 15 November 2025)).

Overall, EGCG reduced CRC cell viability, proliferation and migration, while inducing apoptosis. These effects were associated with increased Bax, AMPK activation, caspase-3/-9 activation, cleaved PARP, and E-cadherin, along with reduced MMP activity, Akt, Bcl-2, and STAT3 signaling (Figure 3).

### 2.2. EGCG Encapsulated in Nanoformulations Against Colorectal Cancer: In Vitro Studies

Improving the oral bioavailability, metabolic stability, chemical stability, and tumor-specific accumulation of EGCG may further enhance its clinical potential [19,22]. Delivery systems such as polymeric nanoparticles, liposomes, micelles, and ligand-targeted nanocarriers have been developed to improve EGCG stability, cellular uptake, sustained release, and tumor-directed delivery [48].

WGA-EG-NP nanoparticles containing EGCG, 5-FU, gelatin, chitosan, and wheat germ agglutinin (WGA) improved drug uptake, cytotoxicity, and apoptosis in HT-29 and CT-26 CRC cells (Table 2). HPLC showed 1.07–2.10-fold higher uptake with WGA-EG-NP than other formulations, with the greatest uptake at 50 mg/L EGCG and 150 mg/L 5-FU. WGA-EG-NP produced the strongest viability reduction, and in CT-26 cells WGA-EF-NP treatment produced the highest early, late, and total apoptosis, while NP, WGA, and WGA-NP alone did not induce apoptosis or necrosis. These findings indicate that co-loading of EGCG with 5-FU in WGA-conjugated nanoparticles leads to greater CRC cell drug uptake and improved efficacy for both agents [49].

Wang et al. [50] treated CT-26 and HCT116 CRC cells with EGCG–manganese combination nanoparticles (MnEGCG). MnEGCG reduced viability more strongly than EGCG alone in a heat-, concentration-, and time-dependent manner. MnEGCG reduced the expression of HSP90, a molecular chaperone that facilitates the folding and stabilization of oncogenic proteins, as well as intracellular ATP levels and the expression of the anti-apoptotic genes XIAP, VCP, and SIL1. Conversely, MnEGCG increased ROS generation and the expression of the pro-apoptotic genes EIF2AK3, ATF6, and XBP1. MnEGCG triggered pyroptosis in gasdermin (GSDMD)-overexpressing CT-26 and HCT116 cells, as indicated by increased GSDMD-N fragment formation and elevated expression of pyroptosis-related genes, including caspase-1, AIM2, and NLRP3, along with enhanced release of IL-1β, LDH, and ATP. These findings suggest that MnEGCG induces an immunogenic inflammatory form of CRC cell death through activation of the caspase-1/GSDMD pyroptosis pathway [50]. EGCG-PEGMA-MAA-NPs were designed for colon-targeted delivery and increased cytotoxicity 92-fold over 72 h compared with free EGCG in HT-29 CRC cells. DAPI and AO/EtBr staining showed greater nuclear damage and apoptosis, while DCFH-DA and Rh-123 staining showed increased ROS and reduced mitochondrial membrane potential. EGCG-PEGMA-MAA-NPs also induced apoptosis on a larger scale compared to free EGCG and caused G2/M accumulation. Overall, pH-sensitive PEGMA-MAA nanoparticles enhanced EGCG delivery and anticancer effects [51].

EGCG-loaded nanoparticle formulations were examined and compared with free EGCG in HCT116, HT-29, HCT15, and HEK293 cells. PLGA-PEG-FA-EGCG (PPF-E) had greater encapsulation efficiency, drug loading, and slower in vitro EGCG release than PLGA-EGCG (P-E) and PLGA-PEG-EGCG (PP-E). PPF-E showed the highest cytotoxic efficacy, followed by PP-E, P-E, and free EGCG. In folate receptor (FR)-positive HCT116 and HT-29 cells, flow cytometry and confocal microscopy showed higher EGCG release and uptake with PPF-E than PP-E or P-E, likely due to high FR expression. All formulations increased DNA fragmentation in HCT116 cells. PPF-E exhibited the strongest apoptotic effect by reducing Bcl-2 and elevating caspases 3 and 9, Apaf-1, cytochrome c, and Bax [52].

**Figure 4:**
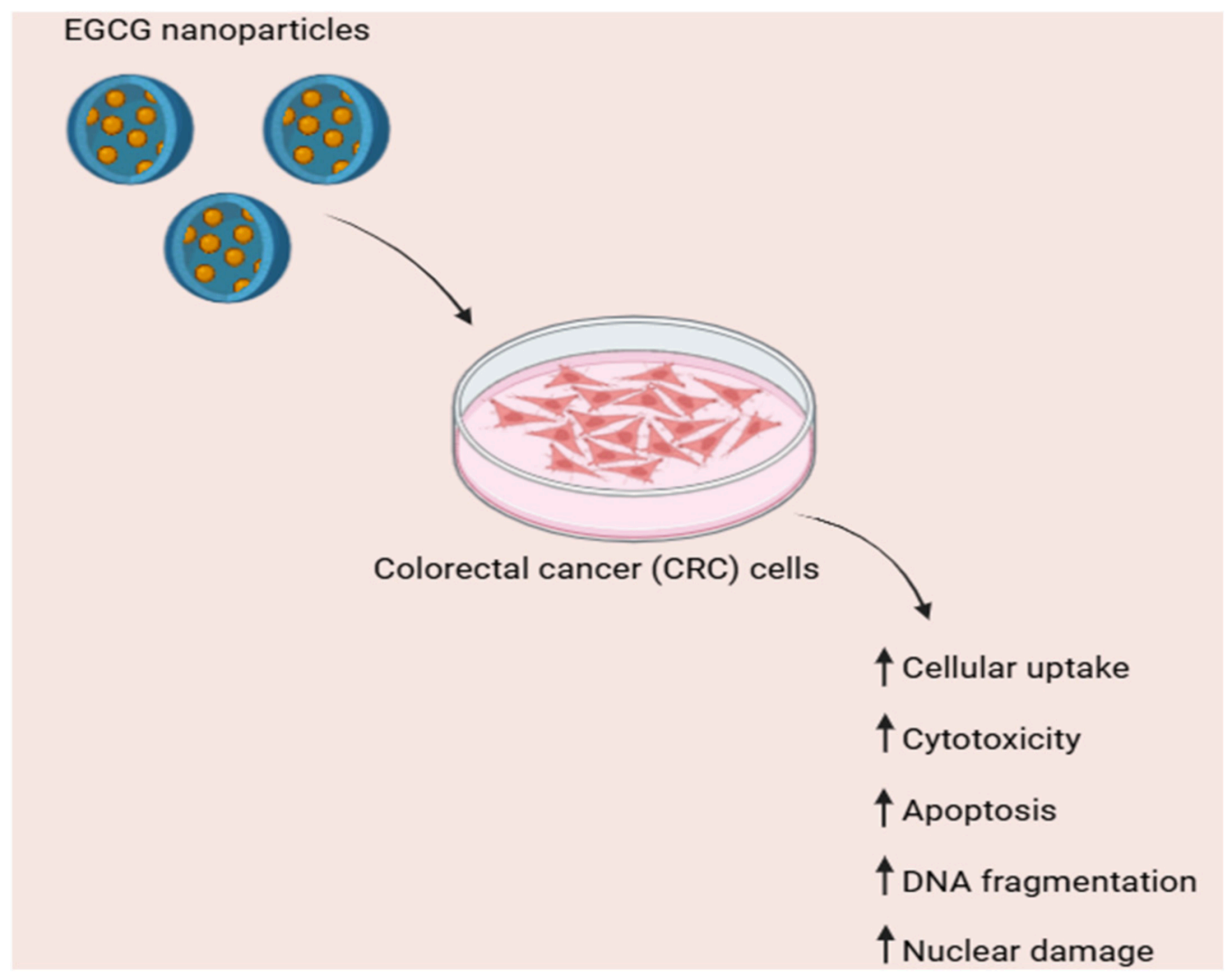
Summary of the effects of EGCG incorporated into nanoparticles in human colorectal cancer cells. EGCG nanoformulations showed increased cellular uptake, apoptotic effects, cytotoxicity, DNA fragmentation, and nuclear damage. ↑ increased. Created in Biorender. (Achilleos, N. (2026). https://BioRender.com (accessed on 15 November 2025)).

Taken together, the current data on EGCG nanoformulations suggest that encapsulation of EGCG in nanoparticles is a promising strategy for CRC treatment, as it enhances tumor drug uptake and potentiates the cytotoxic and pro-apoptotic effects of EGCG, including DNA fragmentation and nuclear damage (Figure 4).

### 2.3. EGCG Combined with Other Therapeutic Approaches Against Colorectal Cancer: In Vitro Studies

EGCG alone or combined with panaxadiol (PD) reduced proliferation and increased apoptosis and cell cycle arrest in HCT-116 and SW480 CRC cells (Table 3). After 48 h, EGCG, PD, and EGCG-PD reduced proliferation dose-dependently, with the strongest suppression seen using EGCG-PD. EGCG induced G2/M arrest, while EGCG-PD decreased S-phase duration. EGCG-PD also induced apoptosis in HCT-116 cells, more than PD alone [53].

Hu et al. [54] found that EGCG alone or combined with oxaliplatin or cisplatin reduced viability and induced autophagy in DLD-1 and HT-29 CRC cells. EGCG plus oxaliplatin and EGCG plus cisplatin produced the strongest viability reduction and showed synergistic effects indicated by a combination index (CI) < 1. EGCG alone and combination treatments increased LC3-I-to-LC3-II conversion, autophagosome formation, and acidic vesicular organelle formation, indicating autophagic cell death. Autophagy inhibition reduced combination-induced viability loss, suggesting that autophagy contributed to EGCG–platinum cytotoxicity. Collectively, these data indicated that EGCG synergizes the therapeutic effect of cisplatin and oxaliplatin through the autophagic pathway in human CRC cells [54].

In HCT-115, HCT-116, and HT-29 cells, EGCG combined with 5-FU reduced viability without affecting healthy HLF cells. Cells were treated with EGCG, 5-FU, or 5-FU plus EGCG. EGCG reduced viability concentration-dependently, with the strongest effect observed with combination treatment. In HCT-116 cells, EGCG treatment reduced sphere formation and the expression of the liver metastasis marker CD133 and the cancer stem cell (CSC) self-renewal regulator Nanog. EGCG also reduced the mRNA levels of the drug resistance-associated transporters ABCC1 and ABCG2, inhibited the proliferation-promoting Nek2/Akt signaling pathway, and induced apoptosis. The G1-phase cells decreased, while the S-phase cells increased after EGCG treatment. Collectively, these findings suggest that EGCG suppresses CRC stemness and metastatic potential [55].

EGCG enhanced 5-FU cytotoxicity and apoptosis in DLD1 and HCT-116 CRC cells. EGCG-5-FU significantly reduced viability in DLD1 and HCT-116 cells compared to 5-FU alone. Combination treatment significantly inhibited colony formation and induced apoptosis more than 5-FU alone in both cell lines. These effects were associated with increased cleaved caspase-3, cleaved PARP, and Bad; decreased Bcl-2 and MDR1; and increased miR-155-5p. EGCG also activated NF-κB and suppressed GRP78, while GRP78 overexpression reversed EGCG-mediated 5-FU sensitization. These data suggest that EGCG enhances the sensitivity of CRC cells to 5-FU through GRP78/NF-κB/miR-155-5p/MDR1 pathway inhibition [56].

Jin et al. [57] found that EGCG inhibited proliferation, migration, invasion, and viability while inducing apoptosis in HCT116, SW480, and CaCo2 CRC cells. EGCG reduced viability dose-dependently, most significantly in SW480 cells, without affecting normal NCM460 colon cells. In SW480 cells, EGCG reduced proliferation, with 40 μM EGCG exhibiting greater antiproliferative activity than 5-FU alone. Both EGCG and 5-FU increased apoptosis and mitochondrial membrane potential collapse through decreasing Bcl-2 and increasing Bax expression. EGCG also inhibited migration and invasion by increasing E-cadherin and decreasing N-cadherin levels and suppressing Sonic Hedgehog (Shh) and PI3K/Akt signaling through the downregulation of PI3K, p-Akt, Smo, and Gli-1. Activation of Smo or PI3K partially reversed the effects of EGCG, whereas inhibition of these pathways produced similar antitumor effects. Collectively, these findings suggest that EGCG induces apoptosis and inhibits proliferation by suppressing the Shh and PI3K/Akt signaling pathways in CRC cells [57].

In normal endothelial cells (NECs) exposed to HT-29 conditioned medium (CM), combined curcumin and EGCG reduced viability, migration, invasion, tube formation, and angiogenesis. HT-29 CM promoted NEC transition into tumor endothelial cells (TECs) along with JAK/STAT3 activation and increased IL-8. Curcumin–EGCG treatment decreased the mRNA expression of STAT3, JAK, and IL-8; reduced the protein levels of p-JAK, p-STAT3, and IL-8; and downregulated the angiogenesis-associated markers TEM1, TEM8, and VEGFR2. These findings suggest that EGCG, in combination with curcumin, may disrupt angiogenic signaling between CRC cells and endothelial cells through inhibition of the IL-8/JAK/STAT3 axis [58].

EGCG combined with radiotherapy reduced cell growth and colony formation and increased apoptosis and autophagy more than either treatment alone in HCT-116 CRC cells. After 2 weeks, combination treatment saw the greatest reduction in colony number compared with either treatment alone. Combination treatment also significantly reduced viability more strongly than monotherapy. These effects were associated with increased Nrf2 nuclear translocation and higher LC3 and caspase-9 mRNA expression, suggesting enhanced autophagy and apoptosis. This study suggests that EGCG enhances radiation sensitivity in CRC cells through Nrf2 activation and autophagy induction [59].

Wu et al. [60] found that EGCG combined with irinotecan caused greater DNA damage, cell cycle arrest, apoptosis, and autophagy and reduced proliferation, migration, and invasion in RKO and HCT116 CRC cells more than either treatment alone. The combination synergistically (CI < 0.7) reduced proliferation and migration in an EGCG dose-dependent manner, and invasion was only inhibited in HCT116 cells. EGCG enhanced irinotecan-induced DNA damage in HCT116 cells, likely through increased topoisomerase I inhibition. EGCG also enhanced irinotecan-induced cleavage of ATM and p-ATM, promoted S- and G2-phase arrest in RKO and HCT116 cells respectively, and reduced the expression of the cell cycle regulators cyclin D1, cyclin B1, and CDK4. In addition, EGCG also increased autophagic vacuoles and LC3B-II-to-LC3B-I transformation, suggesting that autophagy enhanced irinotecan-induced apoptosis. Collectively, these findings indicate that EGCG–irinotecan combination therapy enhances apoptosis in CRC cells by triggering DNA damage responses, cell cycle arrest, and autophagy [60].

EGCG alone or with irinotecan increased mitochondrial dysfunction and apoptosis while reducing intracellular ROS and GRP78 membrane translocation in RKO and HCT116 CRC cells. The irinotecan and EGCG combination produced the highest mitochondrial depolarization and induced apoptosis in both cell lines. ROS reduction was greatest with the combination, dose-dependent, and was associated with decreased levels of Bcl-2 and increased levels of cleaved PARP. EGCG also reduced irinotecan-induced GRP78 membrane translocation and increased intracellular GRP78 accumulation, suggesting enhanced ER stress. These data indicate that EGCG enhances the chemosensitivity of CRC cells to irinotecan through GRP78-mediated ER stress [61].

In HT-29, HCT-116, and SW480 CRC cells, EGCG combined with TRAIL significantly reduced viability, increased apoptosis, and increased the sub-G1 population. EGCG plus TRAIL increased TUNEL-positive cells, cleaved PARP, and reduced pro-caspase-8 more than EGCG alone. EGCG and TRAIL also decreased DR5 receptor and mRNA levels, while DR5 siRNA reduced combination cytotoxicity and lowered sub-G1 accumulation. Collectively, these findings illustrate that EGCG increases TRAIL sensitivity of CRC cells through activation of the pro-apoptotic caspase 8 and DR5 [62].

### 2.4. EGCG Against Colorectal Cancer: In Vivo Animal Studies

In SW837 xenograft mice, EGCG in drinking water suppressed tumor growth and decreased VEGFR-2, p-VEGFR-2, p-Akt, and p-ERK (Table 4). RT-PCR also showed decreased VEGF mRNA levels, suggesting reduced VEGF/VEGFR-associated angiogenic signaling [63].

In SCID mice xenografted with RKO cells, EGCG reduced liver metastatic area and angiogenesis while increasing apoptosis in liver metastases (Table 4). Immunohistochemistry showed suppressed tumor growth, TUNEL assay showed increased apoptosis, and anti-CD31 staining showed reduced angiogenesis. These findings suggest that EGCG may reduce CRC liver metastasis, although further validation is needed [34].

Jin et al. [58] administered EGCG, curcumin, or their combination in CRC PDX mice every other day for 4 weeks. EGCG and curcumin each reduced tumor growth, but the combination produced the strongest effect. Combination treatment also reduced angiogenesis more than either agent alone, shown by decreased microvessel density and hemoglobin content. These effects were associated with stronger suppression of p-JAK, p-STAT3, and IL-8. Collectively, these data indicate that EGCG inhibits cell growth and angiogenesis through inhibition of the JAK/STAT3/IL-8 pathway, an inflammation-associated signaling axis [58].

In female FVB/N mice, EGCG reduced azoxymethane/dextran sulfate sodium (AOM/DSS)-induced ACF, colitis, tumor load, tumor size, and malignant colonic tumor formation. Mice were assigned to control, AOM/DSS, or AOM/DSS plus EGCG groups. EGCG significantly reduced ACF incidence and colonic adenocarcinoma incidence. EGCG partly restored microbiome changes by increasing Clostridiaceae and Lactobacillus, which are associated with anticancer effects, while attenuating the increased levels of pro-inflammatory Bacteroides bacteria. EGCG’s reduction of colitis and partial restoration of pro-inflammatory microbiota changes supports its potential to attenuate inflammation-linked tumor development. More studies are required to elucidate the role of the human microbiome in CRC development [62].

EGCG plus 5-FU reduced tumor growth more than vehicle or either treatment alone in BALB/c mice xenografted with DLD-1 cells. Mice received EGCG, 5-FU, or EGCG plus 5-FU for 14 days. Tumor volume reduction was greatest in the combination group. EGCG alone and the combination treatment also reduced MDR1 and increased miR-155-5p, suggesting improved 5-FU sensitivity through reduced MDR1-mediated chemoresistance [56].

WGA-EF-NP reduced tumor volume, increased tumor inhibition rate (TIR), and improved pharmacokinetics and biodistribution in orthotopic CT-26 xenograft mice. Mice were assigned to NP, WGA, WGA-NP, EGCG, 5-FU, EGCG-NP, 5-FU-NP, EF-NP, WGA-5-FU-NP, or WGA-EF-NP groups. After 16 days, WGA-EF-NP produced the greatest tumor volume reduction and the highest TIR, followed by WGA-5-FU-NP, while EGCG alone had the lowest TIR. H&E and TUNEL staining showed necrosis and increased apoptosis, with minimal systemic toxicity. WGA-EF-NP increased EGCG half-life, 5-FU half-life, and produced the highest EGCG and 5-FU distribution in colon and tumor tissue. Overall, these data suggest that co-loading of EGCG and 5-FU in WGA-conjugated nanoparticles shows improved antitumor effects in vivo [49].

In SW480 xenografted BALB/c nude mice, EGCG reduced tumor weight, invasion, and growth while inducing apoptosis. EGCG and 5-FU both significantly decreased tumor weight, but only 5-FU reduced body weight. EGCG treatment induced apoptosis by increasing Bax and decreasing Bcl-2 and inhibited tumor invasion by increasing E-cadherin and decreasing N-cadherin expression. EGCG also suppressed PI3K/Akt and Shh signaling, which are associated with proliferation, shown by decreased PI3K, p-Akt, Smo, and Gli-1 [57].

Khiewkamrop et al. [42] found that in mice xenografted with HCT-116 CRC cells, EGCG reduced tumor volume and weight with antitumor efficacy similar to 5-FU, but without weight loss, cellular damage, or morphological changes. EGCG decreased Bcl-2 and increased cleaved caspase-3, BNIP3, and Bak, indicating apoptosis induction. EGCG also reduced free fatty acids, de novo lipogenesis, ATP, p-Akt, and p-ERK1/2, suggesting suppression of PI3K/Akt/mTOR-related lipid metabolism. Overall, these findings suggest that EGCG triggers apoptosis by suppressing de novo lipogenesis, potentially through inhibition of the PI3K/Akt/mTOR pathway [42].

EGCG reduced tumor volume, tumor formation rate, and aberrant crypt foci (ACF), while increasing tumor inhibition rate in DMH-induced CRC SPF Wistar rats. Rats received saline, DMH, or DMH plus EGCG. Moderate- and high-dose EGCG reduced tumor volume, while high-dose EGCG significantly reduced ACF. EGCG decreased tumor formation rates dose-dependently. Tumor inhibition also increased dose-dependently at week 20. Using network pharmacology analysis, the authors further linked EGCG’s anti-tumor effects to CRC-related pathways that included IL-6 and IκB/NF-κB signaling, suggesting that inflammatory pathway modulation may contribute to its activity [64].

Afzal et al. [65] showed that EGCG reduced membrane damage, inflammation, goblet cell disintegration, and mucosal injury while increasing antioxidant enzyme activity in DMH-induced CRC Wistar rats. Rats received PBS, DMH, DMH plus EGCG, or EGCG alone. EGCG significantly reduced malondialdehyde and increased SOD, catalase, GSH, GST, GPX, and GR only in DMH-treated rats, indicating increased antioxidant activity. EGCG also reduced goblet cell disintegration and decreased DMH-induced inflammation, as evidenced by decreased levels of NF-κB, COX-2, and IL-6. Collectively, these data suggest that EGCG reduces inflammation and mucosal injury and ameliorates colon toxicity in rats with DMH-induced CRC [65].

EGCG plus irinotecan reduced tumor mass more than either monotherapy in BALB/c mice xenografted with HCT116 cells. Immunohistochemistry showed that combination treatment decreased Ki67 and increased GRP78 and caspase-3 more strongly than EGCG or irinotecan alone, indicating reduced proliferation and increased apoptosis/ER stress [61].

In luciferase-labeled CT-26 xenograft mice, MnEGCG nanoinhibitors increased tumor uptake, reduced tumor growth and mesenteric dissemination, increased survival, induced pyroptosis, and enhanced tumor immune activation. Biodistribution analysis showed higher tumor accumulation of DiR-labeled MnEGCG than DiR alone, with similar radiance in other organs. MnEGCG treated at 43 °C produced the strongest tumor inhibition and showed the greatest HSP90 inhibition, pyroptosis induction, reduction in mesenteric dissemination, and survival prolongation. MnEGCG-HIPEC also increased CD3, IFN-γ, TNF-α, and IL-6, suggesting enhanced T-cell infiltration and conversion of cold to hot tumors [50].

EGCG promoted dendritic cell (DC) phagocytosis, remodeled the tumor microenvironment, and enhanced anti-CTLA-4 therapy in CT26 tumor models. In BALB/c mice xenografted with EGCG-treated CT26 cells, EGCG increased tumor-free survival. EGCG increased bone marrow-derived DC phagocytosis of CT26 cells. In CT26 tumors, EGCG increased DC infiltration, CD80 expression, and IFN-γ/TNF-α production by tumor-infiltrating CD8+ T cells. EGCG alone did not reduce tumor size, but EGCG plus anti-CTLA-4 antibody reduced tumor size more than anti-CTLA-4 alone. These findings indicate that EGCG increases the immune response, leading to improved anti-CTLA-4 antibody in vivo efficacy against CRC [46].

In CW-2 xenografted mice, EGCG reduced tumor growth compared with the control. EGCG did not negatively affect pain, behavior, body weight, or survival. The tumor-suppressing effects combined with the minimal toxicity of EGCG suggest that EGCG is effective against CRC and well tolerated in vivo [47].

**Figure 5:**
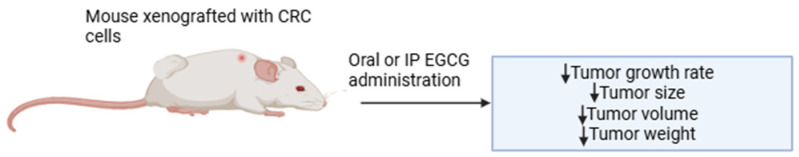
EGCG reduced tumor growth in animals xenografted with human CRC cells. ↓: reduced. Created in Biorender. (Achilleos, N. (2026). https://BioRender.com (accessed on 15 November 2025)).

Overall, oral or intraperitoneal EGCG administration reduced CRC tumor growth rate, size, volume, and weight in xenograft and chemically induced CRC animal models (Figure 5).

### 2.5. EGCG Against Colorectal Cancer: Human/Clinical Studies

In a randomized clinical trial, patients received two 500 mg green tea extract (GTE) tablets daily, each containing 225 mg GTE and 51.5 mg EGCG (Table 5). After 1 year, GTE reduced metachronous polyps and metachronous adenomas compared to the placebo group. Relapsed adenomas and recurrent polyps were also lower with GTE. GTE was well tolerated, with no serious adverse effects, although alanine aminotransferase (ALT) increased up to twice the normal range. Body weight, body mass index, waist circumference, fasting glucose, lipid levels, and C-reactive protein (CRP) were unchanged [66].

In a randomized phase II placebo-controlled clinical trial, patients with current or prior advanced colorectal adenomas or cancer received polyphenon E (Poly E) at 600 mg twice daily containing approximately 780 mg EGCG. Poly E did not significantly change rectal aberrant crypt foci (ACF) number, ACF burden, or overall adenoma recurrence. However, Poly E significantly reduced recurrence of adenomas ≥5 mm compared to the placebo group. Post-study surveillance showed fewer recurrent adenomas with Poly E than placebo and longer median time-to-adenoma recurrence. Poly E was well tolerated without increased adverse effects [67].

In the randomized, double-blind, placebo-controlled, multicenter MIRACLE trial, green tea extract (GTE) did not significantly reduce metachronous adenomas in the total population or females in either modified intention-to-treat (mITT) or per-protocol (PP) analyses. In males, GTE reduced metachronous adenomas in both mITT and PP populations. GTE did not reduce serrated or advanced adenomas but decreased nonadvanced adenomas in males. High-grade intraepithelial neoplasia/CRC was also lower with GTE than placebo, with CRC developing in one GTE patient and two placebo patients [68].

**Figure 6:**
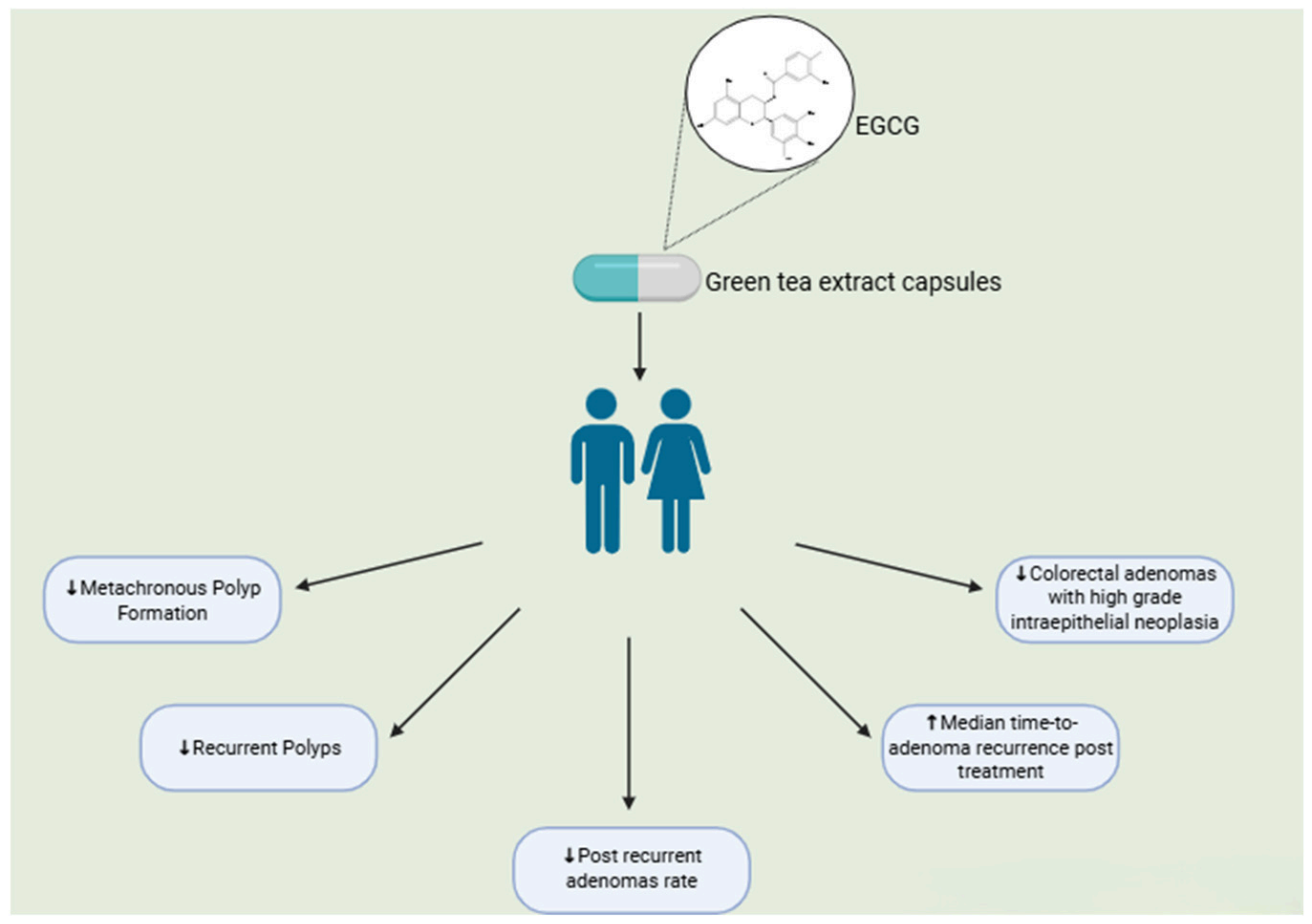
Summary of the effects of green tea extract (GTE) capsules with predetermined EGCG concentration in clinical trials. GTE decreased metachronous polyp formation, recurrent polyps, post-recurrent adenoma rate, and colorectal adenomas with high-grade intraepithelial neoplasia and increased median time-to-adenoma recurrence post-treatment.↑ increased, ↓ reduced Created in Biorender. (Achilleos, N. (2026). https://BioRender.com (accessed on 15 November 2025)).

Overall, GTE decreased metachronous polyp formation, recurrent polyps, post-recurrent adenoma rate, and colorectal adenomas with high-grade intraepithelial neoplasia and increased median time-to-adenoma recurrence post-treatment (Figure 6). However, all clinical trials used GTE and none of them used EGCG alone. Therefore, more clinical trials need to be conducted with purified EGCG alone to determine its effects in CRC patients.

## 3. Discussion

CRC remains a major global cancer burden, ranking third in incidence and second in mortality in 2020 [1]. Although surgery, chemotherapy, monoclonal antibodies, and radiotherapy are used clinically, treatment toxicity, cost, and resistance continue to drive interest in complementary therapeutic strategies. This review summarized studies examining the anticancer effects of the green tea catechin EGCG in CRC.

In vitro studies consistently showed that EGCG reduced CRC cell proliferation, viability, and migration while increasing apoptosis (Table 1, Figure 3). These effects were mainly associated with inhibition of PI3K/Akt/mTOR, STAT3, and Bcl-2 signaling, along with increased Bax, Bad, caspase activation, cleaved PARP, and AMPK activation. EGCG also reduced STAT3/CXCL8 signaling and NET formation in neutrophil–CRC cell models, indicating effects on inflammation-associated tumor microenvironment signaling [43]. EGCG concentrations ranged from 2 to 1000 μM and from 5 to 1000 μg/mL.

EGCG nanoformulations improved cellular uptake and cytotoxicity compared with free EGCG. PPF-E was particularly effective in folate receptor-positive HT-29 and HCT116 cells, while WGA-EF-NP, EGCG-PEGMA-MAA-NP, and MnEGCG formulations produced stronger apoptosis induction and viability reduction than regular EGCG [49,50,51,52]. MnEGCG also activated caspase-1/GSDMD-associated pyroptosis, linking this nanoformulation to inflammation-related CRC cell death [51]. EGCG also showed synergistic effects with irinotecan, supporting its potential as a chemotherapy-sensitizing agent (CI < 0.7) [60].

It should be noted that the EGCG concentrations employed in in vitro studies exhibit substantial variability (0.05–1000 μM), with many investigations using concentrations of 100–1000 μM that are unlikely to be achieved in vivo following dietary consumption or standard oral supplementation because of EGCG’s limited absorption, rapid metabolism, and low bioavailability. Therefore, although these in vitro studies have been instrumental in identifying the molecular targets and signaling pathways through which EGCG inhibits CRC cell proliferation, induces apoptosis, and suppresses metastasis, their direct clinical relevance remains uncertain.

CRC animal models treated with EGCG exhibited reduced tumor growth rate, size, volume, and weight (Table 4, Figure 5). Tumor analyses showed increased pro-apoptotic, anti-migratory, and anti-proliferative markers, along with reduced expression of molecules involved in proliferation, migration, apoptosis evasion, angiogenesis, and inflammatory signaling. EGCG reduced NF-κB, COX-2, IL-6, colitis severity, and pro-inflammatory microbiota changes. The animal studies provide an insight into the in vivo properties of EGCG and stronger evidence of the therapeutic properties of EGCG against CRC than in vitro studies. However, the range of doses used in animals (5–200 mg/kg) and the effects observed against CRC might not translate into clinical benefit in humans.

Clinical trials using green tea extract (GTE) or Polyphenon E preparations with a defined EGCG content showed potential benefit in CRC prevention. These studies reported reductions in metachronous adenomas, recurrent/metachronous polyps, and relapsed adenomas [66,67,68]. However, all clinical trials used GTE and none of them used purified EGCG alone, and some endpoints, including rectal ACF number and advanced adenoma occurrence, were not significantly improved [67,68].

It should be noted that clinical studies evaluating EGCG as a standalone intervention are virtually nonexistent. To date, the limited number of available clinical trials have primarily investigated green tea extract (GTE), which contains EGCG along with other catechins and bioactive constituents, rather than purified EGCG alone. Consequently, the specific therapeutic efficacy, optimal dosing, and safety profile of EGCG cannot be clearly distinguished from those of the other components present in GTE.

Additional in vivo investigations and well-controlled human clinical studies are required to establish the pharmacokinetic profile of EGCG, determine the systemic and colonic tissue concentrations that can realistically be achieved, and define the doses necessary to elicit clinically meaningful therapeutic benefits. These studies will be essential for translating the compelling mechanistic evidence into effective EGCG-based strategies for CRC prevention and treatment.

Overall, preclinical and clinical findings are encouraging, but current evidence remains incomplete. Current studies suggest that EGCG can modulate inflammatory and immune-related pathways in CRC models, although these effects remain less characterized than its effects on proliferation, apoptosis, and tumor growth.

Other phytochemicals, including curcumin, resveratrol, quercetin, and sulforaphane, have also been studied in CRC and other cancers and share overlapping anticancer mechanisms, including effects on proliferation, apoptosis, inflammation, oxidative stress, angiogenesis, and treatment sensitization [69,70,71,72,73,74,75]. However, the present review focused specifically on EGCG, and direct comparisons across phytochemicals remain limited by differences in models, doses, formulations, and outcome measures.

Few in vivo studies and no clinical trials have directly examined EGCG–chemotherapy combinations. Future studies should prioritize purified EGCG, EGCG nanoformulations, and EGCG–chemotherapy combinations to clarify EGCG’s efficacy, bioavailability, toxicity, and molecular mechanisms in CRC, including comparative and combination studies with other well-studied phytochemicals.

## 4. Conclusions

EGCG shows anticancer activity against CRC by reducing cell viability, proliferation, migration, tumor growth, and tumor burden while promoting apoptosis. In vivo studies support EGCG-mediated reductions in tumor volume, mass, and growth rate, and clinical studies suggest that EGCG-containing extracts may reduce adenoma and polyp recurrence. EGCG nanoformulations are especially promising because they improve cellular uptake and cytotoxicity. More clinical studies are needed to define the therapeutic role of EGCG and EGCG nanoformulations in CRC.

## Figures and Tables

**Table 1:** Effects of epigallocatechin-3-gallate against colorectal cancer cells: in vitro studies.

Cell Line	Dose/Duration	Effects	Mechanism	Level of Evidence	Treatment Type	Reference
HCT-116	EGCG 0.05, 0.1, 0.5, 1, 5, 10 μM	↓ Cell viability ↓ Cell invasion ↓ Cell growth ↓ Cell proliferation	↓ p-Met ↓ p-Akt ↓ p-Erk	Preclinical (in vitro)	Purified EGCG	[32]
HT-29 LoVo SW480 HCT-8	EGCG 10, 20, 35 μg/mL	↓ Cell proliferation ↑ Apoptosis ↑ Cell cycle arrest	↓ HES1 ↑ Notch1 ↓ Notch2	Preclinical (in vitro)	Purified EGCG	[33]
HCT-116 RKO	EGCG 12.5, 25, 50, 100 μM	↓ Cell proliferation ↑ Apoptosis ↓ Cell viability	↓ p-AKT ↑ p-p38 ↓ VEGFR2 mRNA	Preclinical (in vitro)	Purified EGCG	[34]
DLD-1 SW480	EGCG 20, 40, 60 μM (DLD-1) EGCG 10, 20, 40 μM (SW480)	↓ Cell proliferation ↑ Apoptosis ↓ Spheroids	↓ p-GSK3β ↑ GSK3β ↓ β-catenin ↓ c-Myc ↓ Cyclin D1 ↓ PCNA ↓ Bcl-2 ↑ Bax ↑ Caspase 8 ↑ Caspase 9 ↑ Caspase 3 ↓ CD133 ↓ CD44 ↓ ALDHA1 ↓ Oct-4 ↓ Nanog	Preclinical (in vitro)	Purified EGCG	[35]
HT-29 3T3	EGCG 125, 250, 500, 1000 μM	↓ Cell viability ↑ ER stress ↑ Apoptosis	↑ BiP ↑ PERK ↑ p-eIF2α ↑ ATF4 ↑ IRE1α ↑ Caspase 3/7	Preclinical (in vitro)	Purified EGCG	[36]
HT-29	EGCG 88.1 μM	↑ Iron chelation	↑ TfR ↓ FtH	Preclinical (in vitro)	Purified EGCG	[37]
HT-29	EGCG 10, 20, 30, 40, 50, 60 μg/mL	↓ Cell proliferation ↑ Apoptosis ↑ G1-phase arrest ↑ Autophagy ↑ Gene level alterations	↑ p53 ↑ p21 ↓ Cyclin D1 ↑ Caspase-3 ↑ Caspase-9 ↑ LC3B ↑ Beclin-1 ↓ IFI6 ↓ XAF1 ↓ PTEN ↓ VEGFA ↑ AKR1C1 ↑ TMEM97 ↑ IL-8	Preclinical (in vitro)	Purified EGCG	[38]
SW480 SW620 LS411N	EGCG 25, 50 and 100 μg/mL (SW480) EGCG 50, 100 and 200 μg/mL (SW620 and LS411N)	↓ Cell proliferation ↑ Apoptosis ↑ Mitochondrial-membrane potential disruption ↓ Cell migration (SW480)	↑ Cleaved caspase-3 ↑ Cleaved PARP ↓ STAT3 and pSTAT3 ↓ Bim, Bcl-2, MCL-1 ↑ Vimentin ↓ E-cadherin	Preclinical (in vitro)	Purified EGCG	[39]
HCT116 HT-29 3T3-L1	EGCG 40, 60, 80, 100 μM	↓ Cell viability ↓ Lipid droplet formation ↓ Triglyceride contents ↓ De novo fatty acid synthesis ↓ Cellular respiration ↓ Glycolysis ↑ Fatty acid metabolism regulation	↓ FASN ↓ ACLY ↓ ACC ↓ SCD1 ↓ SREBP1c ↓ CPT1A ↓ ACOX1 ↓ PPARα ↓ CD36/FAT ↓ FATP4 ↑ ATGL ↑ HSL ↓ PGC-1α ↓ UCP1 ↓ ECAR ↓ OCR ↑ p-AMPK	Preclinical (in vitro)	Purified EGCG	[40]
HCT116 HT-29	EGCG 25, 50 or 100 μM	↓ Cell proliferation ↓ Cell migration ↓ CAF capabilities	↓ CAFs’ lactate levels ↓ CAFs’ PFK ↓ CAFs’ MCT4	Preclinical (in vitro)	Purified EGCG	[41]
HCT116 HT-29	EGCG 0.2, 0.4, 0.6, 0.8 and 1 mM Palmitate 100 μM	↓ Cell proliferation ↑ Apoptosis ↑ Mitochondrial membrane potential disruption	↓ MMP levels ↓ ATP levels ↓ De novo lipogenesis (DNL) ↓ ACC expression	Preclinical (in vitro)	Purified EGCG	[42]
SW480	EGCG 5, 10, 25, 50 μM	↓ Cell viability ↓ Cell migration	↓ CXCL8 ↓ STAT3 ↓ MPO ↓ H3Cit	Preclinical (in vitro)	Purified EGCG	[43]
SW620 HCT-116 LoVo RKO	EGCG 25, 50, 75, 100, 125 μM	↓ Cell proliferation ↑ Apoptosis ↑ ROS ↓ Cell migration	↓ Total LATS1/2 ↓ p-LATS1/2 ↑ YAP ↓ p-YAP ↑ YAP nuclear localization ↑ CTGF ↑ CYR61 ↑ ANKRD1 YAP knockdown: ↑ E-cadherin ↑ ZO-3 ↓ N-cadherin ↓Vimentin ↓ Snail ↓ Slug	Preclinical (in vitro)	Purified EGCG	[44]
CaCo2	>10 kDa EGCG polymers 50–1000 μg/mL	↓ Cell viability ↑ Apoptosis	↓ ACE ↓ AT1R ↓ AngII ↑ ACE2 ↑ AGT ↑ Cleaved PARP ↑ Bax ↑ p-p53 ↑ γ-H2AX	Preclinical (in vitro)	Purified EGCG	[45]
HCT116 CT26 HT-29 MC38 SW480	EGCG 50, 100, 200, 400 μM	↑ Apoptosis ↑ Immunogenic cell death ↑ ER stress ↑ Tumor microenvironment alterations	↑ ATF3 ↑ ATF4 ↑ DDIT3 ↑ HMGB1 ↑ CRT ↑ HSP70 ↑ DC infiltration ↑ CD80 ↑ IFN-γ ↑ TNF-α	Preclinical (in vitro)	Purified EGCG	[46]
CaCo2 CW-2 COLO-320 LoVo WiDr	EGCG 5, 10, 20 μg/mL	↓ Cell proliferation ↑ Apoptosis	↑ CCK-18 ↑ TrkB ↓ miR-187-5p	Preclinical (in vitro)	Purified EGCG	[47]

Table legend: ↑ increased, ↓ reduced, p—phosphorylated.

**Table 2:** Effects of EGCG encapsulated in nanoformulations on CRC cells.

Cell Line	Dose/Duration	Effects	Mechanism	Level of Evidence	Treatment Type	Reference
HT-29 CT-26	WGA-EF-NP: 5-FU 0.1, 1, 10, 15, 25, 30, 50, 75, 100, 150, 200 mg/L EGCG 0.033, 0.33, 3.33, 5, 8.33, 10, 16.67, 25, 33.3, 50, 66.67 mg/L	↑ Cellular uptake ↓ Cell growth ↑ Cytotoxicity ↑ Apoptosis	N/A	Preclinical (in vitro)	EGCG nanoformulation	[49]
CT-26 HCT116	EGCG 20, 40, 50, 60, 80, 100 mg/L MnEGCG 12.5, 20, 25, 40, 50, 60, 80, 100 mg/L	↑ Cellular uptake ↓ Heat resistance ↑ Pyroptosis ↓ Cell viability	↓ HSP90 ↑Eif2ak3 ↑ Atf6 ↑ Xbp1 ↓ Xiap ↓ Vcp ↓ Sil1 ↓ intracellular ATP ↑ ROS ↑ GSDMD-N Fragment ↑ Caspase 1 ↑ Caspase 1 gene ↑ Aim2 gene ↑ Nlrp3 gene ↑ IL-1β, LDH, and ATP extracellular leakage	Preclinical (in vitro)	EGCG nanoformulation	[50]
HT-29	EGCG 0.1, 1, 10, 100, 1000 μg/mL EGCG-PEGMA-MAA nanoparticles (NPs) 5 μg	↑ Cytotoxicity ↓ Cell viability ↑ Apoptosis ↑ Nuclear damage ↑ Cell cycle arrest	↑ ROS ↓ MMP	Preclinical (in vitro)	EGCG nanoformulation	[51]
HCT116 HT-29 HCT15 HEK293	EGCG 0–140 μM P-E 0–140 μM PP-E 0–140 μM PPF-E 0–140 μM	↑ EGCG uptake and release ↑ Apoptosis ↑ Cytotoxicity ↑ DNA fragmentation rate	↑ Caspase-3 ↑ Caspase-9 ↑ Apaf-1 ↑ Cyto-C ↑ Bax ↓ Bcl2	Preclinical (in vitro)	EGCG nanoformulation	[52]

Table legend: ↑ increased, ↓ reduced.

**Table 3:** Effects of EGCG combination with other molecules on CRC cells.

Cell Line	Dose/Duration	Effects	Mechanism	Level of Evidence	Treatment Type	Reference
HCT-116 SW480	EGCG 10, 20, 30 μM Panaxadiol 10, 20 μM	↓ Cell proliferation ↑ Apoptosis ↑ Cell cycle arrest	NA	Preclinical (in vitro)	Purified EGCG Combination Treatment	[53]
HT-29 DLD-1	EGCG 25, 50, 100 μM Cisplatin 20 μM Oxaliplatin 20 μM	↓ Cell viability ↑ Autophagy	↑ LC3-I to LC3-II Conversion ↑ Autophagosome formation ↑ Acidic vesicular organelle formation	Preclinical (in vitro)	Purified EGCG Combination Treatment	[54]
HCT-116 HT-29 HCT-115 HLF	EGCG 12.5, 25, 50, 75 μM 5-FU 0.05 μg	↓ Cell viability ↓ Sphere formation ↓ Chemoresistance ↑ Cell cycle profile alterations ↑ Apoptosis	↓ CD133 ↓ Nanog ↓ABCC1 ↓ ABCG2 ↓ Nek2 ↓ Akt	Preclinical (in vitro)	Purified EGCG Combination Treatment	[55]
DLD-1 HCT-116	EGCG 200, 300, 400, 500, 600, 700 μM 5-FU 10, 50, 100, 150, 200, 300 μM EGCG 50 μM + 5-FU 1, 5, 10, 15, 20 or 30 μM	↓ Cell viability ↑ Apoptosis	↑ Cleaved PARP ↑ Cleaved caspase-3 ↑ Bad ↓ Bcl-2 ↓ MDR1 ↑ miR-155-5p ↑ NF-kB ↓ GRP78	Preclinical (in vitro)	Purified EGCG Combination Treatment	[56]
SW480 HCT116 CaCo2	EGCG 20, 40 μM 5-FU 500 μmol/L	↓ Cell proliferation ↑ Apoptosis ↓ Cell migration ↓ Cell invasion ↓ Cell viability ↑ MMP collapse	↑ Bax ↓ Bcl-2 ↑ E-cadherin ↓ N-cadherin ↓ PI3K ↓ p-AKT ↓ Smo ↓ Gli-1	Preclinical (in vitro)	Purified EGCG Combination Treatment	[57]
HT-29 Normal endothelial cells	EGCG 5 μM Curcumin 50 μM	↓ Cell migration ↓ Cell invasion ↓ Angiogenesis	↓ p-JAK p-STAT3 ↓ IL-8 ↓ TEM1 ↓ TEM8 ↓ VEGFR2	Preclinical (in vitro)	Purified EGCG Combination Treatment	[58]
HCT116	EGCG 12.5 μM 2-Gy radiation	↓ Cell proliferation ↑ Apoptosis ↑ Autophagy	↑ Nrf2 nuclear translocation ↑ LC3 ↑ Caspase-9	Preclinical (in vitro)	Purified EGCG Combination Treatment	[59]
HCT116 RKO	EGCG 2, 5, 10, 20, 50, 100, 200 μM Irinotecan 0.2, 0.5, 1, 2, 5 μM	↓ Cell proliferation ↓ Cell migration ↓ Cell invasion ↑ DNA damage ↑ Cell cycle arrest ↑ Apoptosis ↑ Autophagy	↓ Topoisomerase I ↑ ATM cleavage ↑ p-ATM cleavage ↓ Cyclin D1 ↓ Cyclin B1 ↓ CDK4 ↑ Autophagic vacuoles ↑ LC3B II to LC3B I Transformation	Preclinical (in vitro)	Purified EGCG Combination Treatment	[60]
HCT116 RKO	EGCG 20, 50 μM Irinotecan 0.5 μM	↑ Mitochondrial dysfunction ↑ Apoptosis ↓ Intracellular ROS levels ↑ ER stress	↑ Cleaved PARP ↓ Bcl-2 ↑ Intracellular GRP78 ↓ Membrane GRP78	Preclinical (in vitro)	Purified EGCG Combination Treatment	[61]
HT-29 HCT-116 SW480	EGCG 20, 40, 60 μM TRAIL 25, 50 ng	↓ Cell viability ↑ Apoptosis ↑ Sub G1 accumulation	↓ Procaspase-8 ↑ Cleaved PARP ↑ DR5	Preclinical (in vitro)	Purified EGCG Combination Treatment	[62]

Table legend: ↑ increased, ↓ reduced.

**Table 4:** Effects of epigallocatechin-3-gallate against colorectal cancer: in vivo studies.

Animal Model	Dose/Duration	Effects	Mechanism	Level of Evidence	Treatment Type	Reference
BALB/c nude mice xenografted with SW837 cells	EGCG 0.01%, 0.1% in tap water 3 times weekly	↓ Tumor growth	↓ VEGFR2 ↓ p-VEGFR2 ↓ p-Akt ↓ p-Erk ↓ VEGF mRNA	Preclinical (in vivo)	Purified EGCG	[63]
Severe combined immunodeficiency (SCID) mice weighing 21–26 g xenografted with RKO cells	EGCG 30 mg/kg IP every other day	↓ Liver metastatic area ↓ Liver metastatic tumor angiogenesis ↓ Liver metastatic tumor apoptosis	NA	Preclinical (in vivo)	Purified EGCG	[34]
Human colorectal carcinoma PDX mice	EGCG 50 mg/kg Curcumin 50 mg/kg	↓ Tumor growth ↓ Microvessel density ↓ Hemoglobin content ↓ Angiogenesis	↓ p-JAK ↓ p-STAT3 ↓ IL-8	Preclinical (in vivo)	Purified EGCG	[58]
Female FVB/N mice	EGCG 1% (*v*/*v*) 20 mg/kg PO once daily Azoxymethane (AOM) 10 mg/kg IP three times daily + Dextran Sodium Sulfate (DSS) 2.5% (*v*/*v*) for three consecutive days after injection	↓ Aberrant crypt foci (ACF) ↓ Colitis ↓ Malignant colonic tumors ↓ Tumor load ↓ Tumor size	↓ AOM-induced probiotics	Preclinical (in vivo)	Purified EGCG	[62]
Balb/c mice xenografted with DLD-1 cells	EGCG 25 mg/kg 5-FU 20 mg/kg intratumorally	↓ Tumor growth	↓ MDR1 ↑ miR-155-5p	Preclinical (in vivo)	Purified EGCG	[56]
Mice xenografted with CT-26 cells	EGCG 15 mg/kg every 2 days 5-FU 45 mg/kg every 2 days	↑ Biodistribution ↑ Half-life ↓ Tumor volume ↑ Tumor inhibition rate ↑ Necrosis ↓ Systemic toxicity	N/A	Preclinical (in vivo)	Purified EGCG	[49]
BALB/c nude mice	EGCG 20 mg/kg PO 5-FU 50 mg/kg	↓ Tumor weight ↓ Tumor growth ↑ Apoptosis ↓ Tumor invasion	↑ Bax ↑ E-cadherin ↓ Bcl-2 ↓ N-cadherin ↓ PI3K ↓ p-AKT ↓ Smo ↓ Gli-1	Preclinical (in vivo)	Purified EGCG	[57]
HCT-116-xenografted nude mice	EGCG 30, 50 mg/kg IP 5-FU 20 mg/kg	↓ Tumor growth rate ↓ Tumor volume ↓ Tumor weight ↑ Apoptosis	↑ DNA breaking ↑ Cleaved caspase-3 ↑ BNIP3 ↑ Bak ↓ Bcl-2 ↓ ATP ↓ DNL activation ↓ p-Akt ↓ p-ERK1/2	Preclinical (in vivo)	Purified EGCG	[42]
SPF Wistar rats with dimethylhydrazine (DMH)- induced CRC	Normal saline twice weekly DMH 40 mg/kg twice weekly EGCG 50, 100, 200 mg/kg PO once daily	↓ Tumor volume ↓ Aberrant crypt foci (ACF) ↓ Tumor formation rate ↑ Tumor inhibition rate	N/A	Preclinical (in vivo)	Purified EGCG	[64]
Wistar rats with dimethylhydrazine (DMH)- induced CRC	EGCG 10, 20 mg/kg IP DMH 40 mg/kg	↓ Membrane damage ↓ Inflammation ↓ Mucosal damage ↓ Goblet cell disintegration ↑ Antioxidant activity	↓ DMH ↑ SOD ↑ Catalase ↑ GSH ↑ GST ↑ GPX ↑ GR ↓ NF-kB ↓ IL-6 ↓ COX-2	Preclinical (in vivo)	Purified EGCG	[65]
Balb/c xenografted with HCT116 cells	EGCG 5 mg/kg IP Irinotecan 4 mg/kg	↓ Tumor mass	↓ Ki67 ↑ GRP78 ↑ Caspase-3	Preclinical (in vivo)	Purified EGCG	[61]
Female Balb/c xenografted with luciferase-labeled CT-26 cells	MnEGCG 50 mg/L IP	↑ Tumor uptake ↓ Tumor growth ↑ Tumor inhibition rate ↑ Pyroptosis ↑ Survival ↓Tumor mesentery dissemination ↑ T-cell tumor infiltration ↑ Immune response ↓ Normal tissue toxicity	↓ HSP90 ↑ IFN-γ ↑ TNF-α ↑ IL-6 ↑ +CD3	Preclinical (in vivo)	Purified EGCG	[50]
BALB/c mice xenografted with CT26 cells (EGCG- and not EGCG-treated) Bone marrow cells from BALB/c mice cultured with EGCG-treated CT26 cells	EGCG 400 μM EGCG 30 mg/kg IP Anti-CTLA4 antibody 200 μg	↑ Tumor-free survival ↑ DC phagocytosis ↑ T-cell activation ↓ Tumor size ↑ DC infiltration ↑ CD8+ T cells	↑ CD80	Preclinical (in vivo)	Purified EGCG	[46]
BALB/c mice xenografted with CW-2 cells	EGCG 50 μg/kg IP	↓ Tumor size	N/A	Preclinical (in vivo)	Purified EGCG	[47]

Table legend: ↑ increased, ↓ reduced, p—phosphorylated.

**Table 5:** Clinical trials examining the effects of green tea extract (GTE) on CRC patients.

Subjects	Dose/Duration	Effects	Level of Evidence	Treatment Type	Reference
Humans (*n* = 143) Patients who had undergone endoscopic polypectomy for the complete removal of colorectal adenomas	Green tea extract (GTE) 450 mg (103 mg EGCG) twice daily PO	↓ Metachronous polyps formation ↓ Recurrent polyps ↓ Metachronous adenomas Formation ↓ Relapsed adenomas	Randomized Clinical Trial (Level II)	Green Tea Extract	[66]
Humans (*n* = 39) History of current or prior advanced colorectal adenomas or cancer	Polyphenon E (65% EGCG) 600 mg twice daily PO	→ % change in rectal ACF number → ACF burden → General adenoma recurrence rate ↓ Adenoma recurrence rate for polyps ≥5 mm ↑ Median time-to-adenoma recurrence post-treatment ↓ Post-recurrent adenomas rate	Randomized Clinical Trial (Level II)	Green Tea Extract (Polyphenon E)	[67]
Humans (*n* = 879) Patients between 50 and 80 years old with ≥1 histologically confirmed colorectal adenoma(s) removed within 6 months before recruitment during a complete colonoscopy with no remaining colorectal adenomas	GTE capsules (EGCG 150 mg per capsule) 1 capsule twice daily for 3 years	→ Detactable metachronous colorectal adenomas in females ↓ Detactable metachronous colorectal adenomas in males → Advanced colorectal adenomas → Nonadvanced colorectal adenomas in females ↓ Nonadvanced colorectal adenomas in males → Serrated colorectal adenomas ↓ Colorectal adenomas with high-grade intraepithelial neoplasia/CRC	Randomized Clinical Trial (Level II)	Green Tea Extract	[68]

Table legend: ↑ increased, ↓ reduced.

## Data Availability

This is a review article. No new data were created in this article.
